# The Impact of the Daily Mile™ on School Pupils’ Fitness, Cognition, and Wellbeing: Findings From Longer Term Participation

**DOI:** 10.3389/fpsyg.2022.812616

**Published:** 2022-04-28

**Authors:** Josephine N. Booth, Ross A. Chesham, Naomi E. Brooks, Trish Gorely, Colin N. Moran

**Affiliations:** ^1^Moray House School of Education and Sport, University of Edinburgh, Scotland, United Kingdom; ^2^Faculty of Health Sciences and Sport, University of Stirling, Scotland, United Kingdom; ^3^School of Health, Social Care and Life Sciences, Centre for Health Sciences, University of the Highlands and Islands, Inverness, United Kingdom

**Keywords:** physical activity, wellbeing, schools, children, cognition

## Abstract

**Background:**

School based running programmes, such as The Daily Mile™, positively impact pupils’ physical health, however, there is limited evidence on psychological health. Additionally, current evidence is mostly limited to examining the acute impact. The present study examined the longer term impact of running programmes on pupil cognition, wellbeing, and fitness.

**Method:**

Data from 6,908 school pupils (mean age 10.2 ± 0.7 years), who were participating in a citizen science project, was examined. Class teachers provided information about participation in school based running programmes. Participants completed computer-based tasks of inhibition, verbal and visual-spatial working memory, as well as the Children’s Feeling scale and Felt arousal scale to determine subjective wellbeing. A multistage 20-m shuttle run test was used to estimate fitness.

**Results:**

From our total sample of 6,908 school pupils, 474 participants had been taking part in a running programme for <2 months (Shorter term participation); 1,004 participants had Longer Term participation (>3 months); and 5,430 did not take part in a running programme. The Longer Term participation group had higher fitness levels than both other groups and this remained significant when adjusted for age, sex and SES. Moderated regression analysis found that for the Shorter Term participation group, higher shuttle distance was associated with better visual-spatial working memory. Effect sizes were small though.

**Conclusion:**

We identified small and selective positive impact of participation in school based running programmes on fitness and cognition. While no long term benefit was identified for cognition or wellbeing, the impact on fitness and short term benefit suggest schools should consider participation.

## Introduction

Recent reviews have concluded that greater physical activity is associated with improved cognition and academic attainment in young people (Barbosa et al., 2020; Chaput et al., 2020). Previous research has demonstrated positive longitudinal associations between physical activity and both academic attainment (Singh et al., 2012; Booth et al., 2014) and cognition (Booth et al., 2013), however, evidence from acute studies and interventions is equivocal (Daly-Smith et al., 2018; Singh et al., 2018). Fitness is also thought to have a positive role in the relationship between physical activity, cognition and attainment and has been suggested as a possible explanatory mechanism (Donnelly et al., 2016). However, levels of physical activity are low globally (Aubert et al., 2021) and so efforts to support increases are warranted.

Classroom physical activity breaks are increasing in popularity in the United Kingdom (for a discussion see Routen et al., 2017). Generally, classroom activity breaks involve short bouts of physical activity during or between academic lessons (Daly-Smith et al., 2018) which occur in addition to break/recess time and aim to increase physical activity and disrupt sedentary behaviour. A vast number of schools are adopting these programmes, however, there are limitations in the scientific evidence base for their effectiveness with many studies viewed as low quality (Daly-Smith et al., 2018). The health benefits of these breaks are no doubt a key consideration, however, it is also important to determine any impact on academic attainment and underlying cognitive skills as these classroom activity breaks often take time away from academic lessons.

The Daily Mile^TM^^1^, is an example of a classroom activity break which is gaining popularity; children run/walk outside for approximately 15 min each school day. Pupils can determine the pace themselves, they can take part in their regular school clothes and should not need to change into sports outfits, and teachers are advised it should take place during curricular time on at least 3 days of the week. It is estimated that over 13,000 schools across 88 countries take part in The Daily Mile™ (“The Daily Mile, 2021, Accessed 21 October 2021”). This differs from other school based running programmes, such as Marathon Kids (^2^ Chalkley et al., 2018) and the Golden Mile^3^ in that it occurs during class time and is a break from class activity, not an addition to existing break or lunch times (Ryde et al., 2018).

Despite its widespread adoption and positive perception amongst teachers (Malden and Doi, 2019), there is limited quantitative evidence surrounding the impact of The Daily Mile™ on a variety of pupil outcomes. In a quasi-experimental pilot study conducted over 8 months with 391 pupils, The Daily Mile™ was found to lead to increases in moderate-to-vigorous physical activity (MVPA) and fitness, reduction in time spent being sedentary, and improvements in body composition (Chesham et al., 2018). Following this, several studies have examined the impact of The Daily Mile™ on fitness after 3–6 months (Brustio et al., 2019, 2020; de Jonge et al., 2020). To our knowledge, no longer term examination of the impact on pupil fitness has been reported.

It is also vital that the impact that participation in The Daily Mile™ has on pupil cognition and wellbeing be considered, as these are key focusses for pupils, teachers, parents and policy makers. There are inconsistent findings concerning the impact that physical activity breaks or interventions have on pupil cognition (i.e., executive function, IQ) and academic attainment (i.e., results of exams or test performance). While reviews have argued that there is certainly no detrimental impact on academic performance of increasing physical activity in the school setting (Singh et al., 2018), and that there is indeed evidence of beneficial impact (Barbosa et al., 2020), the impact on *cognition* of taking 15 min out of class every day (or 75 min a week) for the school year to complete The Daily Mile™ is not yet known.

In relation to The Daily Mile™, Morris and colleagues examined the acute impact on maths test performance and executive function in a sample of 9 year old children (*n* = 303) (Morris et al., 2019). They reported no benefit for maths test performance or executive function. However, in a large study with over 5,000 children aged approx. 10 years old, an acute beneficial impact has been found for short physical activity breaks including a Daily Mile like activity for aspects of executive function (Booth et al., 2020). Furthermore, a recent study examined the acute impact of participation in The Daily Mile™ in a sample of 104 children (mean age 10.4 years) and found a trend for a positive impact on children’s working memory, but no broad impact on cognition (Hatch et al., 2021). Hatch and colleagues also found that children enjoyed participation and reported a positive impact on social relatedness and autonomy. As studies reported have only examined the acute impact of participation in The Daily Mile™, there is therefore a need to examine these outcomes in relation to longer term participation.

Furthermore, there is widespread evidence demonstrating the positive impact which physical activity has on pupil mental health (Biddle et al., 2019), in particular it is beneficial for reducing the risk of depression (Chaput et al., 2020). In addition, recent research involving 8–12 year olds (*n* = 1,540) found that self-reported physical activity was positively associated with subjective wellbeing and satisfaction with life (García-Hermoso et al., 2020). However, little evidence exists concerning the impact that Daily Mile participation has on pupil wellbeing, although acute benefits on wellbeing have been reported following participation in a Daily Mile like activity (Booth et al., 2020). Given the number of school pupils participating globally, there is therefore an urgent need to understand the longer term impact that participation may have.

One underlying factor which has been proposed to account for the relationship between physical activity and cognition, is physical fitness (Donnelly et al., 2016). However, not all reviews have found supporting evidence for the fitness-cognition hypothesis (e.g., Etnier et al., 2006). Research has found that longitudinal changes in fitness were associated with changes in school attainment in adolescent boys but not in girls (Kyan et al., 2018). However, other research has reported fitness to be positively associated with academic attainment and that the relationship was partially mediated by executive functions (Visier-Alfonso et al., 2020). A review by Ludyga et al. (2016) found that both low and high fit individuals benefit cognitively from an acute bout of physical activity. Despite this, a recent large study on the acute impact of physical activity breaks found that fitness did not mediate the relationship between physical activity and cognition in children (Booth et al., 2020). In addition, fitness has been reported to have an impact on wellbeing in childhood and adolescence (LaVigne et al., 2016; Marques et al., 2017). While taking part in The Daily Mile™ has been found to improve pupils fitness (Chesham et al., 2018; Brustio et al., 2019, 2020; de Jonge et al., 2020) it is important to consider to what extent fitness has an impact on cognition and wellbeing in this context.

The present study will compare groups of pupils who have taken part in The Daily Mile™ for a short time (i.e., 2 months or less) and longer term (i.e., more than 3 months), compared to those who have not participated. The aim is to address gaps in the knowledge base and understand the longer term impact that taking part in The Daily Mile™ has on pupil cognition, wellbeing and fitness, and the relationship between these factors.

## Materials and Methods

### The Present Study

BBC Terrific Scientific^4^ is a project aiming to support young people learning about science through citizen science. Briefly, citizen science characterises generally large scale research studies which involve members of the public in data collection (e.g., Brestovitsky and Ezer, 2019). The BBC Terrific Scientific programme encourages mass participation of school pupils in real world academic research in the United Kingdom. Teachers lead pupils through data collection for research studies employing online resources and lesson plans linked with a United Kingdom University. The pupils involved learn about a range of aspects of research as well as about scientific enquiry. The present study was developed and administered as part of Terrific Scientific and was known as the Exercise Investigation^5^.

### Participants

Participants were volunteers in the BBC Terrific Scientific Programme. 503 class teachers registered their class to take part in the study. Table 1 shows demographic information about the sample. In total, 7,337 children from the registered classes (mean age 10.2 ± 0.7 years; 50% female) provided information on at least one key outcome measurement. Participants were from all parts of the United Kingdom, with 78.1% (*n* = 5,728) being from England, 14% (*n* = 1,024) from Scotland, 7.3% (*n* = 536) from Wales and 0.5% (*n* = 40) from Northern Ireland. Furthermore, according to postcode data, the majority of schools were in the least deprived areas of the country with 14.5% of the sample from Index Multiple Deprivation/Scottish Index Multiple Deprivation 10.

**TABLE 1:** Demographic information: n (%); mean (SD) for age.

Variable	Shorter term participation			Longer term participation			No participation		
	Male	Female	Total	Male	Female	Total	Male	Female	Total
Age (months)	122.29 (9.29)	122.18 (9.39)	122.24 (9.33)	122.61 (8.18)	123.18 (8.22)	122.90 (8.20)	121.70 (8.53)	121.54 (8.56)	121.62 (8.54)
Sex	242 (51.1)	232 (48.9)	474 (6.86)	495 (49.3)	509 (50.7)	1004 (14.53)	2709 (49.9)	2721 (50.1)	5430 (78.60)
**Country**									
England	118 (48.8)	128 (55.2)	246 (51.9)	337 (68.1)	336 (66.0)	673 (67.0)	2241 (82.7)	2249 (82.7)	4490 (82.7)
Scotland	85 (35.1)	76 (32.8)	161 (34.0)	124 (25.1)	122 (24.0)	246 (24.5)	266 (9.8)	252 (9.3)	518 (9.5)
Northern Ireland	0	0	0	9 (1.8)	7 (1.4)	16 (1.6)	12 (0.4)	12 (0.4)	24 (0.4)
Wales	39 (16.1)	28 (12.1)	67 (14.1)	25 (5.1)	44 (8.6)	69 (6.9)	190 (7.0)	208 (7.6)	398 (7.3)
**SES**									
1 (most deprived)	11 (4.5)	11 (4.7)	22 (4.6)	10 (2.0)	9 (1.8)	19 (1.9)	175 (6.5)	143 (5.3)	318 (5.9)
2	49 (20.2)	44 (19.0)	93 (19.6)	46 (9.4)	40 (7.9)	86 (8.6)	203 (7.5)	195 (7.2)	398 (7.3)
3	29 (12.0)	24 (10.3)	53 (11.2)	0	0	0	231 (8.5)	259 (9.5)	490 (9.0)
4	28 (11.6)	28 (12.1)	56 (11.8)	64 (13.1)	50 (9.9)	114 (11.5)	244 (9.0)	217 (8.0)	461 (8.5)
5	29 (12.0)	29 (12.5)	58 (12.2)	100 (20.5)	114 (22.5)	214 (21.5)	176 (6.5)	177 (6.5)	353 (6.5)
6	16 (6.6)	12 (5.2)	28 (5.9)	55 (11.3)	59 (11.6)	114 (11.5)	314 (11.6)	308 (11.3)	622 (11.5)
7	18 (7.4)	15 (6.5)	33 (7.0)	121 (24.8)	112 (22.1)	233 (23.4)	311 (11.5)	292 (10.7)	603 (11.1)
8	20 (8.3)	25 (10.8)	45 (9.5)	4 (0.8)	8 (1.6)	12 (1.2)	320 (11.8)	337 (12.4)	657 (12.1)
9	14 (5.8)	17 (7.3)	31 (6.5)	39 (8.0)	51 (10.1)	90 (9.0)	391 (14.4)	395 (14.5)	786 (14.5)
10 (least deprived)	28 (11.6)	27 (11.6)	55 (11.6)	49 (10.0)	64 (12.6)	113 (11.4)	344 (12.7)	398 (14.6)	742 (13.7)

### Measures

#### Teacher Reported Information

Class teachers provided demographic information when registering their class for the project. They provided information about school postcode which was used to determine IMD/SIMD as an indication of socioeconomic status (SES^6^). Furthermore, they reported pupil year group and whether their class currently took part in a running programme like The Daily Mile™ or something similar. If they responded positively, they were asked the name of the programme, how long they had been participating, and how often their class took part. The core principles of the Daily Mile™ are that participants should take part on at least 3 days of the week and while there is variability reported in schools approach to undertaking this (e.g., Ryde et al., 2018), we used this criteria to clarify participation in line with Brustio et al. (2020).

#### Pupil Reported Demographic Information

Pupils were asked to specify their age and sex when first completing any measurements.

#### Cognition

Cognition was measured using three bespoke computer-based tasks which are described in detail in Booth et al. (2020). Briefly, they involved:

##### Inhibition

Inhibition was measured using an adapted stop-signal task (Logan et al., 1984). Pupils were asked to press a button corresponding to the direction of an arrow. Participants were instructed to suppress their response if the stimuli changed colour (i.e., not press any button). Outcome variables were reaction time (for “go” trials), correct responses, incorrect responses (failure to stop) and an adjusted inhibition score (reaction time for go trials plus number of incorrect responses × 10). Using this method, lower scores equal better performance. The stop-signal task has acceptable reliability and validity in children (Williams et al., 1999) and the use of reaction times adjusted for error rate has been recommended for reaction time tasks (Draheim et al., 2016).

##### Working Memory

Visual spatial working memory was assessed using a computer based adapted version of the static boxes search task (Diamond et al., 1997). Pupils were tasked with searching for a cartoon face hidden in on-screen boxes. Once a face was located, it would not be presented there until the next round. Scores were based on accuracy with an optimum number of presses for the level reached (i.e., for level 4, optimum number of presses = 10) adjusted for the actual number of presses (actual – optimum) so that a lower score indicates better performance.

Verbal working memory was assessed using a reading span task (Daneman and Carpenter, 1980). Pupils were presented with a series of sentences and asked to judge the veracity of the sentence before remembering the last word. The number of sentences presented together increased (i.e., two sentences, then three sentences, etc.) up to a maximum of eight. Scores were based on the total number of words correctly recalled, with higher scores indicating better working memory. This method of scoring has good reliability in adults and children (Friedman and Miyake, 2005; Towse et al., 2008).

#### Subjective Wellbeing

The adapted Children’s Feeling Scale and Felt Arousal Scale (Hulley et al., 2008) were employed to assess the affective component of subjective wellbeing. Children were presented with a Likert scale and pictures with corresponding facial expressions. They were asked: “How do you feel right now?” on a scale of very bad to very good (scored from −5 to +5) and “How awake do you feel right now?” on a scale of very sleepy to very awake (scored from 1 to 6). A higher score indicates greater feelings of wellbeing. This adapted version has been used widely with children (e.g., Budzynski-Seymour et al., 2019; Vazou et al., 2019) and acceptable validity has been reported for single item measures (Van Landuyt et al., 2000).

#### Fitness

Pupils completed the bleep test following standard procedure for the maximal multistage 20-m shuttle run test (Léger et al., 1988). Pupils were grouped into pairs taking it in turns to act as runner and distance recorder before repeating the test with roles swapped. A fuller description is given in Booth et al. (2020). Pupils then entered the number of the level and shuttle which they reached in the online form when they returned to class. Age-corrected VO_2_ max scores were created using procedure described by Léger et al. (1988) following recommendations by Tomkinson et al. (2019).

### Procedure

Ethical permission was granted from the local University ethics committee (UOE ref 1066). Information packs were provided to schools which included health and safety information and information letters for parents. Following British Psychological Society ethical guidance, as Terrific Scientific was deemed an educational activity, parental opt-out consent was employed, in addition to school/teacher consent for class participation. Pupils could, however, choose not to participate in the online measurements. Class teachers were responsible for gathering this information.

Teachers completed an online form to register their class to take part in the BBC Terrific Scientific programme (see text footnote 4). Upon this registration, teachers were given access to a secure website which contained the online measurements. Separate registration information was required from teachers – this ensured that all information held by the research team was independent from information held by the BBC. A set of unique pupil identifiers were computer generated for registered classes and access given to class teachers. Teachers allocated each consenting pupil in their class one of the identifiers and retained this information until the project was complete. Upon completion, teachers were asked to destroy the information linking pupils to identifier information.

Consenting pupils were given access to the online system by their class teacher using the unique identifier. Pupils completed the demographic questions and then tasks of cognition and subjective wellbeing. Lesson plans, videos explaining the tasks and procedure, and downloadable pupil resources were given to class teachers to share with pupils prior to commencing the study (adapted copies accessible from see text footnote 5). Teachers were advised that measurements should not be completed immediately following the pupils arriving in the morning, just after break or lunch time, or just after completing PE (or other PA).

### Statistical Analysis

Outliers were excluded using the inter-quartile rule (i.e., if they were < Q1-1.5*IQR or > Q3 + 1.5*IQR) (Jones, 2019) prior to group categorisation. Residuals and probability plots were inspected to ensure assumptions were met. Missing data was removed using pairwise deletion. Between groups ANOVA was performed to explore differences between participants depending on the duration which they had been taking part in a running programme (longer term and shorter term), as well as those who did not, while controlling for age, sex and SES. Eta-squared (η^2^) estimates of effect size are included and interpreted as small = 0.01, medium = 0.06, and large = 0.14 (Cohen, 1988).

Regression analysis was used to explore the relationship between fitness and outcomes of cognition and wellbeing. Furthermore, model 1 from the PROCESS macro for SPSS (Hayes, 2018) was employed to determine the moderating impact of duration of Daily Mile participation (coded as Shorter term, Longer term, and no participation) on these relationships. All analysis was performed using SPSS (version 25).

## Results

### The Daily Mile™ Participation

From our total sample, class teachers reported that 21.8% (*n* = 1,596) of pupils took part in The Daily Mile™ or similar running programme on at least 3 days of the week, with 799 of these doing it every school day. There were a small number of participants whose teacher reported that they took part in a running programme less than once a week (*n* = 96), or just once a week (*n* = 215). For subsequent analysis, and following Brustio et al. (2020), we excluded participants who were taking part once a week or less, with included participants therefore categorised depending on whether they were taking part in a running programme on 3 or more days, or not taking part at all.

In order to categorise duration of participation, we excluded 118 participants whose teachers reported not remembering when they started doing The Daily Mile™. 29.7% of the sample (*n* = 474) started doing the running programme in the school term in which the study took place, which meant that at the time of data collection, they had been doing it 2 months or less. We categorised this as the Shorter Term participation group. 62.9% (*n* = 1,004) started in the previous school year or earlier, which we termed the Longer Term participation group for analysis. The No Participation group therefore contained the remaining 5,430 participants. Demographic and descriptive statistics for outcome variables for all 6,908 participants included in the analysis can be found in Tables 1–3.

**TABLE 2:** Descriptive data from shuttle run test by country.

Variable	Shorter term participation			Longer term participation			No participation		
	Male (*n* = 242)	Female (*n* = 232)	Total (*n* = 474)	Male (*n* = 495)	Female (*n* = 509)	Total (*n* = 1,004)	Male (*n* = 2,709)	Female (*n* = 2,721)	Total (*n* = 5,430)
**Age corrected VO_2_ max**									
England	47.78 (5.32)	45.92 (4.79)	46.76 (5.10)	48.53 (6.05)	47.06 (5.48)	47.83 (5.82)	48.50 (5.08)	46.88 (4.22)	47.68 (4.73)
Scotland	48.96 (5.55)	45.34 (4.66)	47.18 (5.40)	47.98 (4.81)	47.65 (3.96)	47.81 (4.38)	49.35 (5.40)	46.26 (4.94)	47.79 (5.39)
Northern Ireland	–	–	–	51.88 (6.95)	47.40 (4.16)	49.96 (5.97)	45.10 (1.70)	44.78 (1.67)	44.84 (1.59)
Wales	48.27 (4.14)	42.56 (2.59)	46.37 (4.55)	51.83 (4.60)	46.69 (3.54)	47.97 (4.40)	46.69 (4.50)	45.23 (3.33)	45.94 (4.00)
Total	48.15 (5.24)	45.60 (4.70)	46.84 (5.12)	48.62 (5.65)	47.20 (4.70)	47.88 (5.22)	48.39 (5.10)	46.59 (4.24)	47.47 (4.76)
**Shuttle distance**									
England	608.21 (402.05)	487.06 (369.38)	541.77 (387.64)	724.16 (462.73)	616.09 (432.36)	672.64 (450.61)	699.57 (390.24)	573.48 (312.18)	635.76 (358.37)
Scotland	779.20 (464.56)	537.50 (316.08)	660.82 (413.14)	680.00 (394.51)	633.87 (301.59)	656.17 (348.87)	760.36 (419.01)	537.19 (375.50)	647.79 (412.23)
Northern Ireland	–	–	–	1070.00 (543.20)	760.00 (408.41)	937.14 (480.20)	460.00 (113.14)	464.64 (106.67)	463.64 (101.91)
Wales	722.00 (331.25)	328.00 (142.55)	597.33 (331.13)	948.00 (374.43)	561.33 (229.60)	658.00 (316.71)	584.62 (320.73)	464.00 (245.62)	522.62 (290.29)
Total	667.69 (416.28)	492.37 (348.74)	577.23 (391.86)	730.29 (441.43)	615.51 (363.63)	670.67 (406.40)	693.41 (388.50)	555.32 (314.13)	623.18 (359.25)

**TABLE 3:** Descriptive information from outcome measures [mean (sd)].

Outcome	Shorter term participation	Longer term participation	No participation	Effect size adjusted models (η_*p*_^2^)
				
Affect	2.25 (2.03)	2.20 (2.06)	2.21 (2.01)	0.00
Alertness	4.30 (1.23)	4.36 (1.29)	4.32 (1.23)	0.00
Inhibition: RT adjusted for errors	795.01 (132.51)	802.81 (145.51)	795.84 (138.60)	0.001
Inhibition: mean RT (go trials)	647.65 (124.75)	663.69 (156.76)	641.14 (149.01)	0.003
Inhibition: errors	12.09 (8.56)	11.28 (8.46)	11.73 (8.61)	0.00
Verbal working memory: Total number of words	25.86 (14.80)	26.12 (14.81)	26.09 (14.65)	0.00
Visual-spatial working memory: Actual adj optimum	−41.75 (35.14)	−39.37 (35.02) ***	−43.82 (34.93)	0.002

*p < 0.05; **p < 0.01; ***p < 0.001.

### Impact of Duration of Daily Mile Participation

Between groups ANOVA revealed a statistically significant difference in visual spatial working memory (VSWM) scores in the unadjusted models [*F*(2,6347) = 6.63, *p* = 0.01, η^2^ = 0.002]. Inspection of pairwise comparisons illustrated that the Longer Term participation group had significantly higher scores than the participants who did not participate in the Daily Mile (mean difference = 4.44, SE = 1.25, *p* < 0.001, 95% CI = 1.99 to 6.89). This remained statistically significant when adjustment was made for age, sex, and SES, although the effect size was small (mean difference = 4.50, SE = 1.24, *p* < 0.001, 95% CI = 2.08 to 6.93, η_*p*_^2^ = 0.02).

Furthermore, a statistically significant difference was found between groups for shuttle distance completed [*F*(2,2403) = 4.32, *p* = 0.013, η^2^ = 0.004], and the finding approached conventional levels of statistical significance for Age corrected VO^2^ max [*F*(2,2403) = 2.86, *p* = 0.058, η^2^ = 0.002]. Pairwise comparisons revealed that the Longer Term participation group had greater shuttle distance than the group who did not do the Daily Mile™ (mean difference = 47.49, SE = 21.26, *p* < 0.05, 95% CI = 5.79 to 89.19), as well as those who had Shorter Term participation (mean difference = 93.44, SE = 33.29, *p* < 0.01, 95% CI = 28.27 to 158.59). These associations remained statistically significant after adjustment for age, sex and SES: shuttle distance *F*(2,2391) = 4.76, *p* = 0.009, η^2^ = 0.004; age corrected VO^2^ max *F*(2,2391) = 4.71, *p* = 0.009, η^2^ = 0.004. No further statistically significant differences were found for any outcomes.

### Impact of Fitness on Cognition and Wellbeing and Moderation of Duration

Shuttle distance was entered in a regression model to predict VSWM, as between group differences had been found for this variable. However, no significant model emerged [*F*(1,2506) = 1.03, *p* > 0.05]. Including duration of Daily Mile participation as a moderator of this relationship did lead to a significant model [*F*(5,2369) = 3.35, *p* < 0.001, *R*^2^ = 0.01]. Specifically, VSWM scores differed between the Shorter Term participation group in comparison to no participation, and the resulting interaction term approached conventional levels of statistical significance (see Table 4).

**TABLE 4:** Coefficients from analysis of fitness predicting outcomes, with duration of Daily Mile participation as moderator.

	Affect			Alertness			VSWM		
	Beta	SE	95% CI	Beta	SE	95% CI	Beta	SE	95% CI
Shuttle distance	0.00	0.00	−0.00 to 0.00	0.00	0.00	−0.00 to 0.00	−0.00	0.00	−0.01 to 0.00
Longer term vs. No participation	−0.07	0.21	−0.47 to 0.34	0.04	0.12	−0.20 to 0.28	−3.19	3.65	−10.35 to 3.97
Shorter term vs. No participation	−0.18	0.25	−0.68 to 0.32	0.09	0.15	−0.20 to 0.39	14.44**	**4.57**	**5.48 to 23.39**
Shuttle distance × Longer term	0.00	0.00	−0.001 to 0.001	0.00	0.00	−0.00 to 0.00	0.01	0.01	−0.00 to 0.02
Shuttle distance × shorter term	0.00	0.00	−0.00 to 0.001	−0.00	0.00	−0.001 to 0.00	−**0.01^+^**	**0.01**	−**0.02 to 0.001**
Age corrected VO_2_ max	0.01	0.01	−0.01 to 0.03	0.01	0.01	−0.00 to 0.02	−0.09	0.16	−0.41 to 0.22
Longer term vs. No participation	−0.39	0.99	−2.34 to 1.56	−0.13	0.59	−1.28 to 1.03	−21.68	17.63	−56.26 to 12.89
Shorter term vs. No participation	−1.30	1.31	−3.87 to 1.28	0.41	0.78	−1.11 to 1.93	38.14	23.40	−7.75 to 84.02
VO_2_ max × Longer term	0.01	0.02	−0.03 to 0.05	0.00	0.01	−0.02 to 0.03	0.47	0.37	−0.25 to 1.19
VO_2_ max × shorter term	0.03	0.03	−0.03 to 0.08	−0.01	0.02	−0.04 to 0.02	−0.65	0.50	−1.63 to 0.32

Bold indicates associations of interest. *p < 0.05; **p < 0.01; ***p < 0.001; ^+^p = 0.07.

**FIGURE 1:**
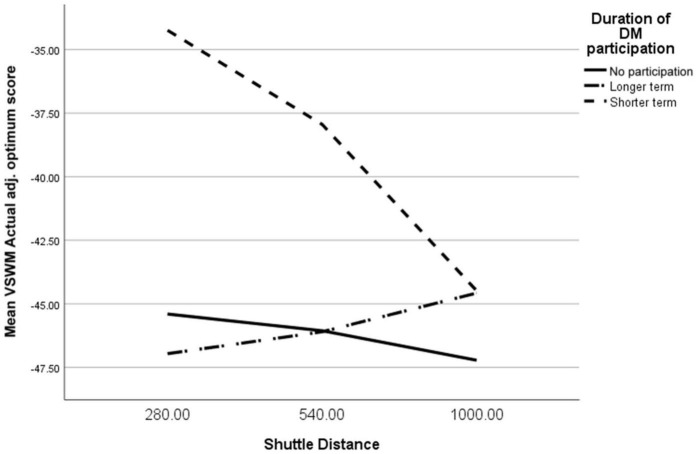
Moderation of Daily Mile™ duration on relationship between shuttle distance and Visual Spatial Working Memory.

Figure 1 illustrates that for the Shorter Term participation group, as shuttle distance increased, VSWM scores decreased, which represents an improvement in VSWM. No significant model was found when age corrected VO_2_ max scores were used as the predictor variable though.

Shuttle distance was also entered as a predictor of alertness and affect, however, no significant model emerged: *F*(1,2537) = 1.01, *p* > 0.05, and *F*(1,2537) = 0.093, *p* > 0.05, respectively. There was also no significant moderation of duration of Daily Mile participation (all *p* values > 0.05). The same pattern emerged when age corrected VO_2_ max was used as the predictor variable – see Table 4 for coefficients.

## Discussion

Overall, we found a small but positive impact of longer term participation in The Daily Mile™ on participants’ fitness. Participants in the present study who had been taking part for 3 months or more, had significantly greater fitness levels than participants who had been taking part for a short term, or who did not participate at all. This was true even when adjusting for confounders. In terms of cognition, we found that the Longer Term participation group had worse performance on the visual-spatial working memory task than participants who were not taking part in the Daily Mile, although the effect size was very small. However, examination of the impact of fitness found that for those who had been participating in The Daily Mile™ for a short term, there was a positive association between fitness levels and visual-spatial working memory performance, whereby increased fitness was associated with better working memory performance. This pattern was not found in the other participant groups though. Furthermore, we found no significant impact of participation on other cognitive outcomes, or on pupils’ wellbeing. Overall findings therefore demonstrate small and selective benefits of taking part in The Daily Mile™.

### Relation to Previous Literature

We found no substantial differences in cognition and wellbeing between those participants who were participating in a running programme at school compared to those who were not. This finding is inconsistent with research demonstrating an acute impact of a Daily Mile like activity (Booth et al., 2020), as well as studies demonstrating long term associations between physical activity and cognition in young people (Booth et al., 2013). Interestingly in Booth et al. (2020) there was no acute impact on visual-spatial working memory but there was a positive impact on other measures of cognition and wellbeing. Similarly, the two other studies which have explored the acute impact of The Daily Mile™ (Morris et al., 2019; Hatch et al., 2021) found varied pattern of results in terms of the acute impact on cognition, although the scale of both of these studies was much smaller. Differential impact of physical activity on tasks of cognition, including working memory, has been reported in previous reviews (Smith et al., 2010) and as the effect size detected in the present study is small, it is worth conducting further research with a range of measures of visual-spatial working memory before strong conclusions are drawn. Our finding adds to the wider evidence base about the impact of classroom activity breaks (Daly-Smith et al., 2018) and suggests that due consideration should be given by researchers to the tasks they use to assess cognition. This aligns with a recent review of the impact of physical education on cognition and academic performance which reported positive associations but cautioned that there were a large number of assessment instruments of varying reliability (García-Hermoso et al., 2021). Thus the extent to which the differential impact of physical activity on cognition is related to the variety of assessment tasks should be considered in future work in this area.

We did find small but statistically significant differences in fitness levels between those who had been taking part in the Daily Mile™ in the longer term, compared to those who did not participate, as well as compared with the Shorter Term participation group. Interestingly, it was the Shorter Term participation group who had the lowest fitness levels from all participants. We were not able to determine whether there were differences in habitual levels of physical activity though and it is possible that pupils who were not taking part in school running programmes were actually doing more MVPA than pupils who were and so their fitness levels were comparable. Participation of pupils in The Daily Mile™ and other running programmes is decided by class teachers and school management and it may be that a needs analysis informs school participation. That is, only where need is perceived to be greatest, do schools choose to participate. Potentially teachers recognised that pupils’ fitness levels were low in the Shorter Term group and so introduced The Daily Mile™ as a mechanism to improve this. This is conjecture, although does align with previous reports (Malden and Doi, 2019).

This possibility is also consistent with evidence showing that uptake of The Daily Mile™ is greater in schools with higher numbers of pupils from disadvantaged backgrounds in England (Venkatraman et al., 2021), where it may be perceived that pupils have less access to other avenues for structured physical activity. Evidence concerning associations of physical activity and SES is inconsistent in primary school aged pupils though (O’Donoghue et al., 2018). Further research should aim to understand factors which influence uptake of running programmes, as well as the longer term impact on pupils.

### Study Strengths and Limitations

The present study has several strengths and provides a unique contribution to our knowledge of this area. This is the first study to consider the longer term impact that taking part in running programmes has on school pupils’ cognitive ability and wellbeing. However, it is important to consider what “longer term” means in the context of the present study. Teachers reported that their class had started doing a running programme in the term of data collection (2 months or less) or in the academic year before or earlier (more than 3 months). We were not able to determine the exact period of participation though. We excluded participants if their teacher could not remember when they started taking part and it is entirely possible that these were pupils who had been taking part for longer periods of time; as we could not verify this, we removed these participants from analysis. As the popularity and duration of uptake of such running programmes increases, it will be important to consider longer term participation in a more refined manner.

Data collection for the present study took place from the middle of August (when schools in Scotland return from summer holiday) until October. As such, some participants took part immediately after the summer holidays. We did not collect data concerning participation in physical activity during the school holidays, and it cannot be ruled out that fitness levels may have been impacted by the break from participation in the Daily Mile, even for those who had been taking part longer term. Unfortunately the citizen science nature of the present study meant we were not able to be flexible with the data collection period. It is important to acknowledge that time of year may have had an impact on the findings of the present study.

One further limitation of the citizen science approach to data collection is that fidelity of measurement for the bleep test is unclear, although the resulting figures for shuttle distance are very similar to those found in a sample of children when collected by trained researchers (e.g., Chesham et al., 2018). This was not an issue for other measurements due to the computerised assessment. We also employed the Léger et al. (1988) equations for estimating VO_2_ max and it must be acknowledged that alternative methods do exist.

Overall, we believe that the benefits of citizen science outweigh the limitations (Den Broeder et al., 2016), however, the variation in running experience between participants must also be acknowledged as a possible limitation.

### Conclusion

We found a positive relationship between longer term participating in The Daily Mile™ and school pupils’ fitness levels. While longer term benefits for cognition and wellbeing were not apparent in this study, the health benefits of physical activity coupled with the acute benefit, which is likely to support learning, makes such physical activity breaks worthwhile and should be considered by class teachers and school management, as well as education policy makers.

## Data Availability Statement

The datasets presented in this article are not readily available because of ethical restrictions. Requests to access the datasets should be directed to the corresponding author.

## Ethics Statement

The studies involving human participants were reviewed and approved by the University of Edinburgh Ethics Committee. Written informed consent from the participants’ legal guardian/next of kin was not required to participate in this study in accordance with the national legislation and the institutional requirements.

## Author Contributions

JB and CM were responsible for data analysis. JB was responsible for interpretation of analysis and for drafted the manuscript and is accountable for all aspects of the work. All authors were responsible for conceptualisation, gaining funding for this research, contributed to critically revising the manuscript, and approved the final version.

## Conflict of Interest

JB and CM currently sit on the research advisory group for The Daily Mile Foundation but did not at the time of study design and data collection. They receive no payment or expenses for this though and their role is to advise concerning research priorities only. The Daily Mile Foundation had no role in the present research. The remaining authors declare that the research was conducted in the absence of any commercial or financial relationships that could be construed as a potential conflict of interest.

## Publisher’s Note

All claims expressed in this article are solely those of the authors and do not necessarily represent those of their affiliated organizations, or those of the publisher, the editors and the reviewers. Any product that may be evaluated in this article, or claim that may be made by its manufacturer, is not guaranteed or endorsed by the publisher.

## Footnotes

1
https://thedailymile.co.uk/

2
https://www.marathonkids.co.uk/

3
http://www.golden-mile.org/

4
https://www.bbc.co.uk/terrificscientific

5
https://www.bbc.com/teach/terrific-scientific/KS2/zmtxy9q

6
https://www.gov.uk/government/statistics/english-indices-of-deprivation-2019
